# Use of Integrated Malaria Management Reduces Malaria in Kenya

**DOI:** 10.1371/journal.pone.0004050

**Published:** 2008-12-30

**Authors:** Bernard A. Okech, Isaac K. Mwobobia, Anthony Kamau, Samuel Muiruri, Noah Mutiso, Joyce Nyambura, Cassian Mwatele, Teruaki Amano, Charles S. Mwandawiro

**Affiliations:** 1 Eastern and Southern Africa Centre for International Parasite Control (ESACIPAC), Kenya Medical Research Institute (KEMRI), Nairobi, Kenya; 2 Centre for Biotechnology, Research and Development (CBRD), Kenya Medical Research Institute (KEMRI), Nairobi, Kenya; 3 African Medical Research Foundation (AMREF), Ministry of Health, Nairobi, Kenya; 4 Division of Vector-Borne Diseases (DVBD), Ministry of Health, Nairobi, Kenya; 5 Environmental Health Program, Department of Epidemiology and Biostatistics and Emerging Pathogens Institute and Whitney Laboratory for Marine Bioscience, University of Florida, St. Augustine, Florida, United States of America; University of Cape Town, South Africa

## Abstract

**Background:**

During an entomological survey in preparation for malaria control interventions in Mwea division, the number of malaria cases at the Kimbimbi sub-district hospital was in a steady decline. The underlying factors for this reduction were unknown and needed to be identified before any malaria intervention tools were deployed in the area. We therefore set out to investigate the potential factors that could have contributed to the decline of malaria cases in the hospital by analyzing the malaria control knowledge, attitudes and practices (KAP) that the residents in Mwea applied in an integrated fashion, also known as integrated malaria management (IMM).

**Methods:**

Integrated Malaria Management was assessed among community members of Mwea division, central Kenya using KAP survey. The KAP study evaluated community members' malaria disease management practices at the home and hospitals, personal protection measures used at the household level and malaria transmission prevention methods relating to vector control. Concurrently, we also passively examined the prevalence of malaria parasite infection via outpatient admission records at the major referral hospital in the area. In addition we studied the mosquito vector population dynamics, the malaria sporozoite infection status and entomological inoculation rates (EIR) over an 8 month period in 6 villages to determine the risk of malaria transmission in the entire division.

**Results:**

A total of 389 households in Mwea division were interviewed in the KAP study while 90 houses were surveyed in the entomological study. Ninety eight percent of the households knew about malaria disease while approximately 70% of households knew its symptoms and methods to manage it. Ninety seven percent of the interviewed households went to a health center for malaria diagnosis and treatment. Similarly a higher proportion (81%) used anti-malarial medicines bought from local pharmacies. Almost 90% of households reported owning and using an insecticide treated bed net and 81% reported buying the nets within the last 5 years. The community also used mosquito reduction measures including, in order of preference, environmental management (35%), mosquito repellent and smoke (31%) insecticide canister sprays (11%), and window and door screens (6%). These methods used by the community comprise an integrated malaria management (IMM) package. Over the last 4 years prior to this study, the malaria cases in the community hospital reduced from about 40% in 2000 to less than 10% by 2004 and by the year 2007 malaria cases decreased to zero. In addition, a one time cross-sectional malaria parasite survey detected no *Plasmodium* infection in 300 primary school children in the area. Mosquito vector populations were variable in the six villages but were generally lower in villages that did not engage in irrigation activities. The malaria risk as estimated by EIR remained low and varied by village and proximity to irrigation areas. The average EIR in the area was estimated at 0.011 infectious bites per person per day.

**Conclusions:**

The usage of a combination of malaria control tools in an integrated fashion by residents of Mwea division might have influenced the decreased malaria cases in the district hospital and in the school children. A vigorous campaign emphasizing IMM should be adopted and expanded in Mwea division and in other areas with different eco-epidemiological patterns of malaria transmission. With sustained implementation and support from community members integrated malaria management can reduce malaria significantly in affected communities in Africa.

## Introduction

The highest burden of global malaria rests upon the population of sub-Saharan Africa which accounts for 80% of the estimated 2 million deaths that occur around the world annually. The reasons for this include the presence of ideal climatic conditions for the breeding of the malaria vector mosquito, the shortage of committed resources and trained personnel, the lack of commitment from governments and communities ravaged by the disease and poor infrastructural capacities for malaria control. The world's most effective malaria vector mosquito species, *Anopheles gambaie* thrives in sub-Saharan Africa where human populations are expanding and ecological conditions are becoming more favorable for malaria parasite transmission. Although malaria puts a heavy toll on human life and on fragile economies of many African countries, recent trends in several countries in Africa are showing encouraging results. In Kenya for instance, there are indications that malaria morbidity and mortality is on a decline as a result of scaled use of insecticide treated nets (ITNs) [1] and increased availability of antimalarial medicines [2]. These recent successes need to be sustained to prevent more deaths and disease.

As advocated by the World Health Organization, the combined use of all the proven tactics and available malaria control tools is the most effective way to check the spread of malaria [3]. These methods include the simultaneous application of a range of malaria control tools targeting malaria *Plasmodium* parasites in humans through case management (CM), the mosquito vector through integrated vector control (IVC) and personal protection (PP). Case management is the use of anti-malaria medicines [4], [5] to remove malaria parasites from the human host to prevent transmission to the mosquito vector, thus preventing the spread of new malaria infections. Integrated Vector Control is the use of adult mosquito killing measures such as indoor adulticide sprays [7] and environmental management to remove the mosquito breeding sites, thus lowering the population densities of malaria vectors. Personal protection includes measures such as insecticide treated nets [6], indoor residual sprays and window and door screens. The use of all these measures together is called Integrated Malaria Management (IMM). Its principle strength is that each individual intervention contributes to the overall reduction of the malaria burden in the population when applied in a practical, economically sustainable manner in the community while protecting the environment. Although the use of ITNs alone has been shown to reduce morbidity and mortality due to malaria [8], [9], there is an additive value when used together with effective antimalarial medicines, indoor residual sprays and environmental management. However, there is a dearth of information on the use of IMM that includes environmental management and its effect on malaria transmission parameters in Kenya and Africa as a whole.

We conducted an entomological study in Mwea division in central Kenya in preparation for malaria control activities. During this study, we noticed that malaria cases in the local referral hospital were declining and sought to find the underlying reasons. In this paper, we analyze the potential factors that may have contributed to the decrease in malaria cases in this community and report that the community has embraced integrated malaria management, which might have contributed to the reduced malaria cases in Mwea division, Kenya.

## Materials and Methods

### Study area

**Figure 1:**
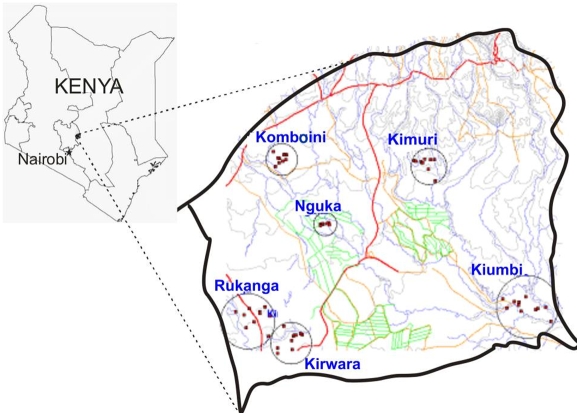
Map of Kenya showing the location of the study area in Mwea division and the study villages. The green marking show the canals running in the rice irrigation paddies. The red lines are the major roadways running through the area and the blue are the several rivers and their tributaries that originate from Mount Kenya and run through Mwea division.

The study was conducted in Mwea Division of Kirinyaga district in Central Kenya (Figure 1) during the period August 2003–July 2004 although the malaria infection data at the local referral hospital was collected until 2007. Mwea Division is located about 100 km northeast of Nairobi, the capital city of the Republic of Kenya. It has an area of 513 square kilometers and a population of 160,000 persons [10]. It lies on the base of Mt Kenya at an altitude of approximately 1200 m above sea level. Several perennial rivers flow through the flat terrain of the poorly drained Mwea division. These kinds of conditions have formed swamps that have led to the development of the largest rice irrigation area in Kenya, known as the Mwea Tebere Rice Irrigation Scheme. The rice irrigation activities in this area produce 90% of the country's rice. The rice irrigation scheme has water canal networks; both planned and unplanned that covers an area of about 13,640 hectares.

**Figure 2:**
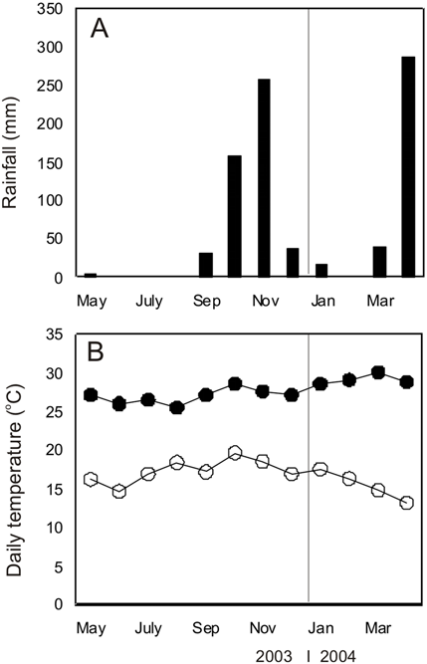
Climate data from the study area in the year 2004–2005. This pattern of climate is very typical in this area and is seen every year.

The ecological and climatic conditions in Mwea are ideal for the breeding of malaria vectors. The mean annual rainfall in this area is in the range of 1200–1600 mm per year and varies by the time of year. The long rains begin usually come from March to June and the short rains from October to December. During the period of study and the preceding years, the rainfall patterns followed the expected pattern described above (Figure 2). Malaria transmission is meso-endemic in this area with *An arabiensis* being the main mosquito species sustaining malaria parasite transmission [11], [12]. *Anopheles funestus* is a minor species also involved in malaria transmission [12], [13]. The entire division has about 23 government and privately sponsored health centers and dispensaries, the largest one of which, the Kimbimbi sub-district hospital, can handle emergency medical care and is the preferred heath centre visited by the people of Mwea division.

### Knowledge, Attitude and Perception (KAP) survey on Integrated Malaria Management

A cross-sectional study was conducted to capture the knowledge, attitude and practices of the community in Mwea division on integrated malaria management (IMM). A structured questionnaire directly addressing IMM usage by the community was administered to 389 households. Study villages and households were selected using a randomized cluster sampling method from a list of households in Mwea division provided by the Central Bureau of Statistics, Ministry of Planning and National Development. These households were physically identified by locally recruited field workers and their locations mapped by a geographical positioning system and the information geo-referenced into the geographic information system (GIS) at Eastern and Southern Africa Centre for International Parasite Control (ESACIPAC) offices in KEMRI. Sensitization and public awareness meetings were held through public meetings (or *barazas*) where the purpose, goals and procedures of the study were explained to the community. The householders were then approached and their consent sought to participate before they were recruited into the study. Data collection was carried out by locally recruited field workers who received training on administration of questionnaire. The questionnaire was pre-tested in a village outside the study sites to prove its effectiveness before it was applied. All the selected households and the owners' names were recorded and assigned identification numbers. The information gathered through the questionnaire included demographic data of the households, knowledge of malaria disease, control measures for malaria and mosquitoes and the cost associated with the treatment of malaria. Household characteristics such as house construction materials, activities of the occupants, environmental factors in homesteads thought to influence human mosquito contact were also collected.

### Estimation of mosquito population densities

The densities of daytime indoor resting anopheline mosquitoes were estimated in 90 households from a sample of 6 villages in Mwea division (Figure 1). Sampling was done over an 8 month period running through the long and short rains. The pyrethrum spray catch method was used to collect indoor resting mosquitoes. The knocked down female anopheles mosquitoes were collected on white sheets and sorted according to the blood meal digestion stage in their midguts. They were preserved for further analysis to identify species and blood meal type ingested using species specific DNA probes and standard enzyme linked immunosorbent assay [14], respectively. The number of household members sleeping in the house the previous night of mosquito collection was also recorded.

### Estimation of malaria transmission rates by mosquitoes

The malaria transmission potential of mosquitoes was determined by estimating the entomological inoculation rate (EIR) in the study villages. The EIR was estimated by multiplying the human biting rate (HBR) and sporozoite rate (SR). The HBR is the mosquito density per house per person multiplied by the proportion of those with human blood in their midgut (Human Blood Index). The SR is the proportion of mosquitoes with sporoziotes in their salivary glands.

### Malaria prevalence in community

Malaria prevalence in the community was assessed by reviewing all the patients' records from the outpatient data at the Kimbimbi sub-District hospital. Consent to view this data was obtained from the District Medical Officer of Health. The Kimbimbi sub-district hospital is the health centre where the majority of the Mwea community seeks treatment. The outpatient records of these patients were collected regardless of age or malaria infection status. From all the outpatient records we only collected laboratory confirmed malaria cases for every month for eight years. The patient details were kept by the health centre although the ESACIPAC malaria team was given access to the data with the permission of the District Medical Officer of Health. The malaria prevalence in the school children was also examined by a one time cross sectional surveillance in public primary schools. Consent to screen the pupils was obtained from parents, guardians and the teachers through Parent Teacher Association meetings that were held prior to the screening. Finger prick blood samples from children were collected and thick blood films made on glass slides. These were later Giemsa stained and examined under a 100× light microscope for malaria parasite identification. For malaria parasite positive samples the species of malaria parasite was determined and the person treated according to the Government policy, through the existing channel at the health centre. The examination of school children and patients records for malaria parasite infection was approved by the KEMRI ethical review committee.

### Sample size determination and statistical analysis

A randomized cluster sampling method was used to select households. Cluster sampling was used because an updated sampling frame was not available. Administrative units called sub-locations within the Mwea division formed the clusters while the primary sampling units were households. To ensure that all the sampled clusters were proportionately represented in the sample, a probability proportional to size of the individual clusters was used to select the number of households. The first household was randomly sampled within the cluster and subsequent households were sampled until the required number of households within that specific cluster was achieved. Consequently, the total number of households required for the calculated sample size was achieved after visiting all the clusters. The sample size (N) of households for the KAP study was determined using the sample size determination equation, where N = *(z^2^pq)/(d)* where *z* = standard normal deviate set at 1.96 corresponding to a 95% confidence level; *p* = a known characteristic of the target population such as prevalence of malaria in the Mwea population which was 39.4%; *q* = 1−*q*; *d* = desired precision level set at 5%. For the entomological studies, the number of sampling villages (N) was determined using the equation (N = *p q N*/(*AE*)*^2^ N+p q*) where *p* = proportion of households with mosquitoes (Because there was no previous information on the proportion of houses (*p*) with mosquitoes in the area, we estimated it to be 50%), *q* = 1−*p*, and the allowable error *(AE)* was set at 5%. A univariate analysis of variance was used to determine the influence of environmental management (clearing of bushes, presence/absence of stagnant water) on indoor resting density, sporozoite rates and EIR in mosquitoes.

## Results

### Community knowledge of malaria disease

A total of 389 households were selected to participate in the KAP study. Ninety eight percent of all the interviewed households reported malaria as the most common disease in the community. Similarly, 98% of the households also knew malaria and that malaria is transmitted by mosquitoes but could not identify which mosquito species was involved. Sixty eight percent identified malaria symptoms including chills (67%), headache (67%) and body pains (66%). Fifty eight percent reported fever (58%), body weakness (53%) and vomiting (37%) as major symptoms of malaria. Thus the community was knowledgeable about the symptoms of malaria. Three months preceding this study, 76% of households reported at least two family members suffering from malaria and within the same time frame, those households experienced malaria between two to four times. Malaria during pregnancy was reported in 4% of households. Malaria related fatalities were reported in 4% of the households over the last five years prior to this study.

### Case management of malaria in households in Mwea division

To control malaria stricken members, households sought treatment from local health centers, either government or privately funded. Ninety seven percent of households reported seeking treatment from the nearest health facility while 3% used medications from local chemists, retail shops and other places. A high proportion of households (81%) used generic drugs before seeking treatment in health centers. Of the households that sought treatment, 52% preferred government health centers over privately funded health centers (22%). Eighty six percent reported using sulphadoxine-pyrimethamine (SP) antimalarial drugs including trade names like Fansidar®, Metakelfin®, Orodor® and Falcidin®; 8% used Chloroquine® and Malaratab® and 6% used pain relievers and other unknown medications. Households reported spending approximately USD 12.00 (equivalent to Kes 840.00 at exchange rate of Kes 70.00 to the dollar) for treatment including diagnosis per single malaria case. The maximum amount of money spent on malaria treatment including associated costs of transport, diagnosis and hospital ward admission was USD 600.00 (∼Kes. 42,000.00 per person). This is a clear illustration of the financial burden imposed by malaria on households. Households that did not get treatment for malaria could not afford to pay for treatment while others were negligent. The average distance a malaria victim travelled to a health center was 2.5 kilometers (approximately 1.55 miles). The nearest health facility was 50 meters away while the farthest was 15 kilometers (9.32 miles) away.

### ITN usage and personal protection measures

About 90% of households interviewed in Mwea division have bed nets, 65% of which are treated with insecticides. This is a very high percentage of bed net and insecticide treated net (ITN) ownership as compared to other areas in the Kenya [15]. The average number of ITNs per household was 2.5±1.3 (range: 1–7 per household). The households reported that the advantages of sleeping under an ITN were to prevent malaria (63.8%) and to reduce the biting nuisance of mosquitoes and other insects. A quarter of the households (26%) used a combination of ITNs and repellants such as mosquito coils, herbs and smoke to protect themselves from malaria and mosquito bites. Almost all the nets were obtained locally within Mwea division from shops and *kiosks*. Twenty two percent of households reported that mobile vendors distributed the ITNs. The Mwea community reported that they have been buying nets from as early as 1981. However, 82% of the households reported that they bought the ITNs between 2000 and 2004. Only 31% of the households reported re-treating the nets using *Powertab*®, a permethrin based product that they bought from the shops. A high proportion of households (97.4%) were willing to buy an insecticide treated mosquito net for USD 3.00 (∼180.00), which was the prevailing selling price of the nets in the area. Only 1% of the households were not willing to buy ITNs because of lack of money. Eighty eight percent of the households bought ITNs to kill and repel mosquitoes and 79% knew that nets should be re-treated after every 5 months. Sixty percent (60%) reported knowing how to treat and re-treat nets. Questioned further, more than 80% of the respondents thought that ITNs were effective in killing and repelling mosquitoes. In homesteads that had only one bed net, respondents reported that women and children should be given preference in using the ITNs. Approximately 40% of the households reported that children were given preference, followed by the father, mother and then youngest child (24%). Eighteen percent of the households reported that the father and mother were third in order of preference in using ITNs. In cases where the father, mother and child are reported as being given preference, it represented cases where the children were too young to sleep on their own and thus slept with their parents.

### Mosquito reduction measures

Households reported that they used a variety of measures to reduce mosquito populations in their homes. Thirty five percent reported that they used environmental management that included removal of stagnant water bodies and removal of brush in the household compound. In addition, the respondents used several methods to lower the numbers of mosquitoes in their homes including mosquito repellants (31%), insecticides canisters (11%) bought from the shops, window and door screens (6%), traditional plants (3%) and “other methods” (3%). These “other methods” include burning herbs in their homes and avoiding construction of homes in swampy areas. Other mosquito reduction measures that were reported in Mwea Irrigation Scheme include anti-larval measures in rice paddies where rice farming households planted a special plant, a water fern that fixes nitrogen in the soil to improve soil fertility of the paddies. Scientifically known as *Azolla* sp., they form a dense canopy over the rice paddies choking mosquito larvae while nourishing the growth of rice. The communities reported that they started using the plant in the year 2000. The communities in Mwea also used intermittent flooding as a way to prevent the emergence of larval mosquitoes. However, we were only able to analyze the effect of removal of water ponds and brush around homes on mosquito density and sporozoites rates.

A univariate analysis of variance was used to analyze the association of environmental management activities such as clearing bushes around the homestead and removing stagnant water bodies that provide ideal mosquito breeding sites on the indoor density of mosquito vectors, the sporozoite rates and entomological inoculation rates in the study villages. In households with stagnant water, a significant association was seen between bush clearing around homesteads and sporozoite rates (P = 0.013) and EIR (P = 0.019) while in households without stagnant water bodies, there was no significant association between clearing the bushes and sporozoite rates (P = 0.124) or EIR (P = 0.448). This suggests that presence of stagnant water was a risk factor for malaria transmission. Overall, there was a significant association between clearing bushes around homesteads and the indoor density of resting mosquitoes regardless of the presence (P = 0.030) or absence (P = 0.038) of stagnant water bodies

### Malaria prevalence in Mwea division

**Figure 3:**
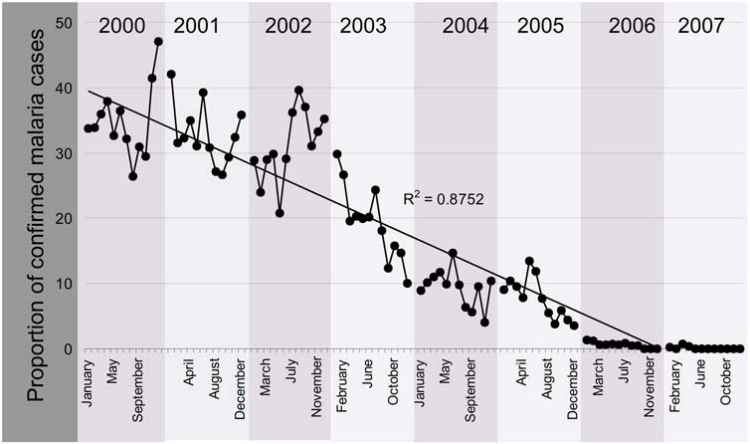
Confirmed malaria cases per 100 persons at the Kimbimbi sub-District Hospital in Mwea division, central province Kenya. The r-square value of the trend line is indicated. The stop gaps in the figure are spaces that separate the years so that the yearly changes in the malaria cases are clearly seen.

At the Kimbimbi sub-district hospital, over the last 8 years (2000–2007), 46,842 individuals representing 29.3% of the population in Mwea division visited this health centre for malaria treatment. Of those examined, 21% (9,900) were positive for either *P. falciparum* (20.05%) or *P. malariae* (0.05%. On average, an equal number of patients visited the hospital every year over the 5 year period but a successive drop in the number of confirmed malaria parasite infections was observed (Figure 3). There was within year seasonal fluctuations in the number of laboratory confirmed malaria cases at the Kimbimbi Health Centre but generally the trend was a steady decline (Figure 3). Peaks of malaria cases that coincided with the seasonal long and short rains were observed indicating climate driven malaria transmission in Mwea division. Even with the seasonal fluctuations, the reduction of malaria was sustained over the 8 years. The malaria infection data from other health centers have not been reported in this paper. In addition 300 school children were selected from 10 primary schools in Mwea division. After getting consent from their guardians and parents, 30 third grade pupils from each of the schools were asked to donate a finger prick blood sample for malaria parasite examination. The mean age of the children was 9.0±2.3 years. There were more girls (54%) than boys (46%) examined in the survey. No malaria parasites were found in the school children.

### Malaria parasite infection in mosquitoes in Mwea division

**Figure 4:**
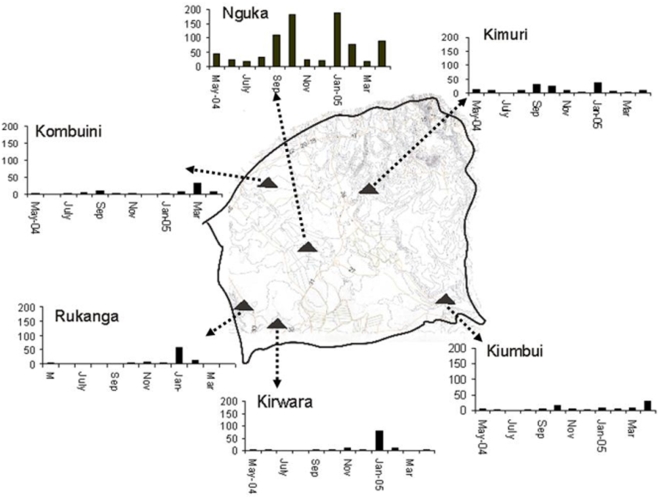
The population density of *Anopheles arabiensis* mosquitoes caught through the 8 month period in Mwea division and is represented by village.

A total of 90 households, 15 homesteads from each of the 6 villages were selected for mosquito collection. The average number of persons sleeping in the households the previous night of sampling was 4.3 in Nguka village, 7.5 in Rukanga village, 5.8 in Kirwara village, 3.2 in Kimuri village, 6.0 in Komboini village and 5.5 in Kiumbui village. A total of 13,620 female *Anopheles* mosquitoes were collected over an 8 month period. Out of these, 13,542 (99.4%) were *An. arabiensis* and only 78 (0.6%) were *An. funestus*. The mosquito population densities remained low throughout the study period (Figure 4) with the exception of one village within the rice irrigation area that contributed 59.6% of all the *Anopheles* mosquitoes caught (Figure 4). The daily daytime resting density of *An. arabiensis* was 66.7 mosquitoes per house in this village while it was 9.0 mosquitoes per house in the other villages outside. The village that contributed the most *Anopheles* mosquitoes was situated within the rice irrigation scheme while the remaining 5 villages that were located outside the irrigation scheme (Figure 4). A high proportion of mosquitoes caught were blood fed (Table 1). High numbers of gravid mosquitoes were also caught (Table 1). Other mosquito species caught included *Anopheles coustani* (5 mosquitoes none was gravid), *Anopheles pharoensis* (6 mosquitoes none was gravid or blood fed), *Anopheles christi* (15 mosquitoes caught with none blood fed, gravid). The rainfall pattern corresponded with peaks of mosquito population abundance, with a 1 month lag time (Figure 2). Monthly rainfall patterns during the period of study obtained from the Kenya Agricultural Research Station based near Kimbimbi hospital in Mwea Division showed two high peaks, one in the month of November 2004 and another one in the month of April 2005 (Figure 2). The maximum and minimum temperatures were stable throughout the study period (Figure 4). The climatic data from the preceding years [16], [17] did not vary or deviate drastically from those recorded during this study period.

**Table 1:** The densities of *An. arabiensis* mosquitoes caught in the study villages in Mwea division and those testing positive for sporoziotes (also expressed as sporozoite rates) is shown.

	Villages sampled in Mwea division					
	Nguka	Rukanga	Kirwara	Kimuri	Kiumbui	Kombuini
No of mosquitoes caught	8,073	970	1,319	1,542	1,042	596
No with sporozoites in salivary gland	11	1	2	3	0	0
Sporozoite rates	0.14	0.1	0.15	0.19	0	0

All other anopheline mosquitoes did not test positive for sporoziotes and are not shown in this table.

### Malaria parasite infection in mosquitoes in Mwea division

**Figure 5:**
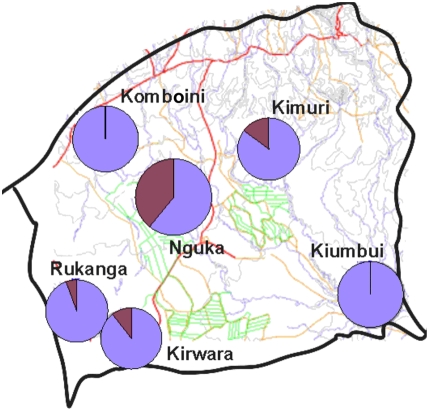
Study villages in Mwea division showing the contribution of each village to the total number of sporozoite positive mosquitoes. The pink part represents the proportion that is infected per village while the blue part represents the total number of sporozoite positive mosquitoes caught in the division.

**Figure 6:**
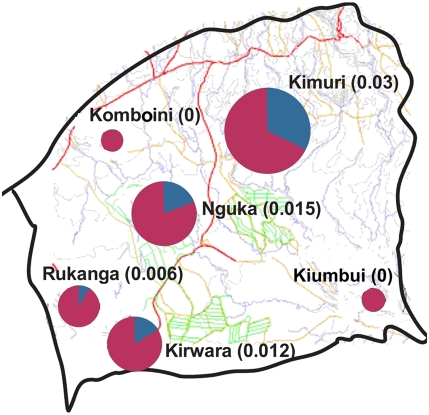
The infectious bites per person per day expressed as entomological inoculation rates (EIR) in the study villages in Mwea division. The size of the circles is proportional to the number of infectious bites received. The blue pie in the circle is the proportion of infectious bites contributed by each village to the total EIR.

A total of 8073 female *An. arabiensis* mosquitoes from the village in the irrigation zone were tested for *P. falciparum* sporozoite infection as compared to 5469 from the other villages outside the irrigation zone. The *An. arabiensis* sporozoite rate in each village was very low (Table 1) whereas no *An. funestus* mosquitoes tested positive for sporozoites (Table 2). Of all the mosquitoes that tested positive for sporozoites the village in the irrigation area (Nguka village) had the highest numbers, followed by Kimuri, Kirwara, and Rukanga (Figure 5). Two villages, Kiumbui and Komboini, did not have sporozoite positive mosquitoes (Figure 5). When the data was grouped together, the overall the sporozoite rate was 0.13%. The human blood index for all the villages was estimated at 0.43. All the 78 *An. funestus* mosquitoes tested negative for sporozoite in the ELISA test. The estimated EIR by village was highest in the non irrigation village of Kimuri (Figure 6). The average EIR for Mwea division was estimated at 0.011 infectious bites per person per day.

**Table 2:** The estimated *P. falciparum* sporozoite rates in *An arabiensis* mosquitoes and their physiological status caught in Mwea division over the study period.

Blood feeding stage	*An arabiensis*					*An funestus*				
	E	BF	HG	G	T	E	BF	HG	G	T
Number of mosquito	1,760	5,095	2,236	4,451	13,542	17	22	12	27	78
Sporozoite positive	5	7	2	3	17	0	0	0	0	0
Sporozoite rate	0.28	0.14	0.09	0.07	0.13	0.00	0.00	0.00	0.00	0.00

## Discussion

This study illustrates that the use of integrated malaria management can effectively reduce malaria transmission in a malaria endemic community Africa. The community members in Mwea division demonstrated good knowledge about malaria disease and how to control it. Through interviews, the community members revealed that they have known and implemented integrated malaria management for several years by using methods that target the malaria parasite in the human host while at the same time implementing methods that shorten the lifespan of the malaria vectors and those that also reduce the malaria vector population thus preventing malaria transmission. These methods as used by the Mwea community constitute integrated malaria management (IMM) and potentially may have led to the reduction in malaria cases at the referral hospital. The effectiveness of IMM in malaria control has a long history and is being advocated as the best way forward because of its success in many tropical environments [3]. One of the key ingredients for its success is the willingness by community members to adopt and apply integrated malaria management tools as a cornerstone in the malaria control efforts [18]. This study has shown that a majority of Mwea community members not only acquired and increasingly used ITNs over the last 4 years, but they also used them in combination with other malaria control and mosquito vector control tools. The impact of this IMM strategy is that it provides the additive effect when all other malaria control tools are used together [19] that has a greater effect in reducing malaria.

Government health centers are a backbone in case management of malaria and need to be strengthened for effective diagnosis and treatment of malaria. Mwea division has a total of 23 health centers funded both by the government and the private sector. Most of the privately funded health centers are missionary funded. These health centers all together serve a population of 160,000 people in the Mwea division. The drug of choice in the treatment of malaria in these health centers has been sulphadoxine-pyrimethamine (SP) until 2004 when this was changed to by the government to Coartem, an artemisinin based combination therapy, owing to increasing resistance levels to SP in the area of close to 30%. However, the change of antimalarial drug policy from SP to arthemether-lumefanthrine only came into effect in health centers across the country in 2006. By the year 2005, the community in Mwea still used SP drugs because Coartem® was not freely provided by the government and some patients could not afford the nominal fee. In the absence of SP the health centre provided amodiaquine® in its place. Although SP is increasingly becoming ineffective due to resistance by malaria parasites in the Mwea area [20], its prophylactic effect could have played a role in the prevention of malaria infection rather than treating actual infected cases prior to 2006. However, recent data in the year 2005, 2006 and 2007 indicates a further downward trend in malaria infection cases at the health centre to almost zero. This could have been largely influenced by the introduction of artemisinin based drugs in the health centre. The prior use of SP drugs and now the use of coartem (arthemether-lumefantrine) together with the use of ITNs are likely to have prevented malaria transmission in the area. Many residents in Mwea bought anti-malarial drugs from local pharmacies and shops when there was a short supply at the health centers. These local shops and pharmacies are also a major avenue for distribution of effective antimalarial drugs [21] should be encouraged.

Even as the usage of ITNs and effective antimalarial medicines is encouraged, the additive value of other control measures on malaria transmission in the community needs to be recognized. The community members in Mwea made use of their knowledge about the benefits of ITNs and antimalarial medicines in the prevention of malaria transmission and treatment of malaria, respectively. The demand for ITNs and free medication provided by the government increased in the community while at the household level it was clear that other malaria control tools were applied to prevent mosquito bites and mosquito breeding. Homesteads that had stagnant water bodies in their compounds were at greater risk of malaria transmission than homes that did not. In addition, removal of bushes around the homesteads may have helped reduce mosquito population in the home. Homesteads reported covering up puddles of water in their homesteads that would provide ideal breeding grounds for mosquitoes and also clearing bushes around the homesteads that provide cover for adult mosquitoes that emerge from the breeding sites. The removal of potential mosquito breeding sites through environmental management could have been motivated by the proximity of this community to the rice irrigation areas which provide breeding grounds for mosquitoes during planting season. Although stagnant water bodies in the households influenced malaria transmission risk, it is also likely that this association may well be attributable to other numerous confounding factors that we were not able to adjust for. The removal of stagnant water was encouraged by public health officials who were targeting bilharzia in the area. This action may have brought collateral benefits by mitigating malaria mosquito breeding.

The use of environmental management has not been advocated strongly as part of an integrated malaria management package. A major advantage of mosquito breeding site removal is that it is cheaper when you consider the cost per unit head of person protected from malaria mosquitoes. A study in Malindi at the coast of Kenya demonstrated the willingness of the community members to use EM to control mosquitoes [22]. So our finding that Mwea residents use environmental management as a method of reducing mosquito populations is very encouraging as it shows that the acceptability of this method for controlling malaria in the community. Considering the level at which ITNs has been publicized in this community and in the country, it is noteworthy that environmental management was also used as a strategy to protect the household from mosquito bites and malaria. This is shows that community members can very readily adopt cheaper mosquito control methods that do not cost the household any money other than their time to prevent transmission of malaria in the household. Although we found an influence of environmental management on malaria transmission risk in households, it should be treated as first step in considering a more elaborate and extensive study to look at the additive effect of environmental management for malaria control and Mwea division in central Kenya may provide an excellent site for this kind of study.

The reduction of malaria cases in the referral hospital in Mwea division over successive years is a reflection of the effectiveness of malaria control measures implemented by this community. Between 1990 and 1995 malaria accounted for 13% of the total deaths reported in Mwea division [10]. However this study found only 4% of the households reporting malaria related deaths. And in another study in Mwea division, it was reported that the malaria prevalence in children was 23.5% [17]. The present study was unable to diagnose any malaria infection cases in the school children, which is a similar age group used by a study in early 2000 [17]. So it is apparent that since the year 2000, there has been a steady reduction of malaria deaths and infection owing to IMM practices of the people. Although it was not until 2004 when the government started an aggressive campaign to expand ITN coverage in Kenya, it is possible that Mwea community may have been already increased their ITN coverage than other areas of the country because of their proximity to irrigation areas where mosquitoes populations are high. Our study also recorded low level sporozoite infection in mosquitoes caught in the villages very similar to those recorded previous studies [11], [12], [17], [23]. Although this may suggest that the situation has not changed from before, it is important to note that reducing parasite prevalence in the human population below 40% would require almost total elimination of low level of sporozoite infections and entomological inoculation rates in mosquitoes [24]. The fact that malaria prevalence has decreased substantially points to consistent and effective control methods that have prevented malaria transmission occurring. That the sporozoite rates and entomological rates have remained low is an advantage because efforts can be focused so that EIR is reduced even further. We were able to show in this study that fewer host seeking mosquitoes (based on presence of human blood in their midguts) harbored sporoziotes suggesting that the community in Mwea embraced personal protection measures that were effective in preventing human-vector contact.

In conclusion, it is possible that the free distribution of ITNs resulted in increased usage and the increased availability of antimalarial medicines played a big role in the malaria decline in this community. In addition, the high knowledge base of the community as it pertains to malaria mosquito control measures may have had a bigger role in the observed low malaria. The introduction of a nitrogen fixing weed into the rice paddies in the early 2000 to boost rice production may also have played a role. However the specific impact of this weed on mosquito population reduction needs to be investigated further. Similarly, a thorough study needs to be initiated to investigate the potential use of community driven larval control activities in the area. The combined usage of a variety of malaria control tools that are widely available together with environmental management measures in Mwea division is likely to have resulted in reduced mosquito populations and malaria transmission in the area.
