# Correction: Use of Integrated Malaria Management Reduces Malaria in Kenya

**DOI:** 10.1371/annotation/e14952c5-b2db-4ff7-976d-6c794d275703

**Published:** 2009-02-04

**Authors:** Bernard A. Okech, Isaac K. Mwobobia, Anthony Kamau, Samuel Muiruri, Noah Mutiso, Joyce Nyambura, Cassian Mwatele, Teruaki Amano, Charles S. Mwandawiro

In the fifth paragraph of the results section the last two sentences contain incorrect data. The correct text should read: In addition, 302 children were selected from one school in Mwea division. After getting consent from their guardians and parents, only 285 pupils from the school were asked to donate a finger prick blood sample for malaria parasite examination. The median age was 7.0 years old (Range: 4½ – 10 years). There were more boys (53%) than girls (47%) examined in the survey. No malaria parasites were found.

